# Impact of pH Modification on Protein Polymerization and Structure–Function Relationships in Potato Protein and Wheat Gluten Composites

**DOI:** 10.3390/ijms20010058

**Published:** 2018-12-24

**Authors:** Faraz Muneer, Eva Johansson, Mikael S. Hedenqvist, Tomás S. Plivelic, Ramune Kuktaite

**Affiliations:** 1 Department of Plant Breeding, Swedish University of Agricultural Sciences, Box 101, SE-23053 Alnarp, Sweden; faraz.muneer@slu.se (F.M.); eva.johansson@slu.se (E.J.); 2 KTH Royal Institute of Technology, School of Engineering Sciences in Chemistry, Biotechnology and Health, Fibre and Polymer Technology, SE-10044 Stockholm, Sweden; mikaelhe@kth.se; 3 MAX-IV Laboratory, Lund University, Box 118, SE-22100 Lund, Sweden; tomas.plivelic@maxiv.lu.se

**Keywords:** wheat gluten, potato protein, chemical pre-treatment, structural profile, tensile properties

## Abstract

Wheat gluten (WG) and potato protein (PP) were modified to a basic pH by NaOH to impact macromolecular and structural properties. Films were processed by compression molding (at 130 and 150 °C) of WG, PP, their chemically modified versions (MWG, MPP) and of their blends in different ratios to study the impact of chemical modification on structure, processing and tensile properties. The modification changed the molecular and secondary structure of both protein powders, through unfolding and re-polymerization, resulting in less cross-linked proteins. The β-sheet formation due to NaOH modification increased for WG and decreased for PP. Processing resulted in cross-linking of the proteins, shown by a decrease in extractability; to a higher degree for WG than for PP, despite higher β-sheet content in PP. Compression molding of MPP resulted in an increase in protein cross-linking and improved maximum stress and extensibility as compared to PP at 130 °C. The highest degree of cross-linking with improved maximum stress and extensibility was found for WG/MPP blends compared to WG/PP and MWG/MPP at 130 °C. To conclude, chemical modification of PP changed the protein structures produced under harsh industrial conditions and made the protein more reactive and attractive for use in bio-based materials processing, no such positive gains were seen for WG.

## 1. Introduction

Wheat gluten (WG) and potato protein (PP) are industrial side-streams originating from wheat and potato processing into ethanol and potato starch, respectively. Both protein-rich by-products are currently mostly utilized in baking (e.g., WG) and animal feed industries (PP). In addition, both of these proteins have attractive properties as raw materials for non-food applications such as in bio-based plastics [1,2]. During the production processes of WG and PP, high drying temperature is utilized, and for PP, an acid mediated coagulation step is also included. Both procedures are known to denature the chemical and structure-determining bonds in the protein and may also result in ionization of amino, carboxyl and phenolic groups [3] and reduced protein functionality [2,4,5]. In addition, for PP being treated at a low pH ≈ 4 during industrial processing, causing definite changes in chemical and physical properties to occur. The chemical processes resulting in intra- and intermolecular changes of the protein limit their processing window, thereby decreasing their suitability for processing into bio-based materials [2,6]. Similarly for WG, the relatively high temperature during drying negatively impacts the proteins and results in aggregated gluten with limited functionality [5]. An alternative and added-value opportunity is to use chemical tools to retain the WG and PP processing ability, both in their present or modified form, for benefit of industry, the consumer and society, thereby contributing to the circular bio-economy. Manipulating the chemical nature of industrial WG and PP would create new possibilities and contribute to the development of alternative uses and an added-value of these proteins for different applications. Development of new bio-based materials from WG and PP proteins is one such promising application.

Un-plasticized WG and PP are difficult to process as their glass transition temperature (*T*_g_) is located close to their thermal degradation temperature [2,7,8,9]. Thus, easy processing requires the addition of chemical agents or polyol-based plasticizers that depress *T*_g_ and widen the processing window [2,4,10,11]. In general, proteins perform better in terms of their structure–functional properties when they are processed at basic pH [12,13]. The basic pH is known to denature and unfold the proteins and thereby expose sulfhydryl and hydrophobic protein sections, which open-up for new protein interactions when processed [14].

Chemical additives, such as NaOH, NH_4_OH or urea, create basic conditions for the protein, resulting in changes of secondary and supramolecular protein structures which correlate with improved functional properties of processed materials [7,9,10,12,13,15,16,17]. However, previous studies have mainly focused on additives or chemical modifiers being added directly to the protein where the blend was immediately processed, thereby leaving a short reaction time for additives to interact with the protein. Although, pre-treatment of the proteins with chemical modifiers in order to modify the protein and later use it for processing has not been evaluated before. This has left a gap for further investigations on the impact of such chemical pre-treatment on protein structure and also functional properties of the final product. In this study, we pre-treated the WG and PP with NaOH in a solution to achieve a basic pH and to promote protein unfolding and induce reactivity. The pH modification was aimed to promote new protein–protein interactions, that could positively impact the mechanical performance of the protein films and composites. The objective was also to understand the effect of the chemical modification on protein molecular and secondary structures, processing behavior and mechanical performance of composites with varying WG and PP ratios. Increased understanding of protein–protein interactions and chemistry, as well as structure-function relationships of proteins in a blend can offer novel opportunities to create versatile and attractive performance of films and composites.

## 2. Results and Discussion

### 2.1. Protein Extractability in pH-Modified Protein Powders, Films and Composites

**Figure 1:**
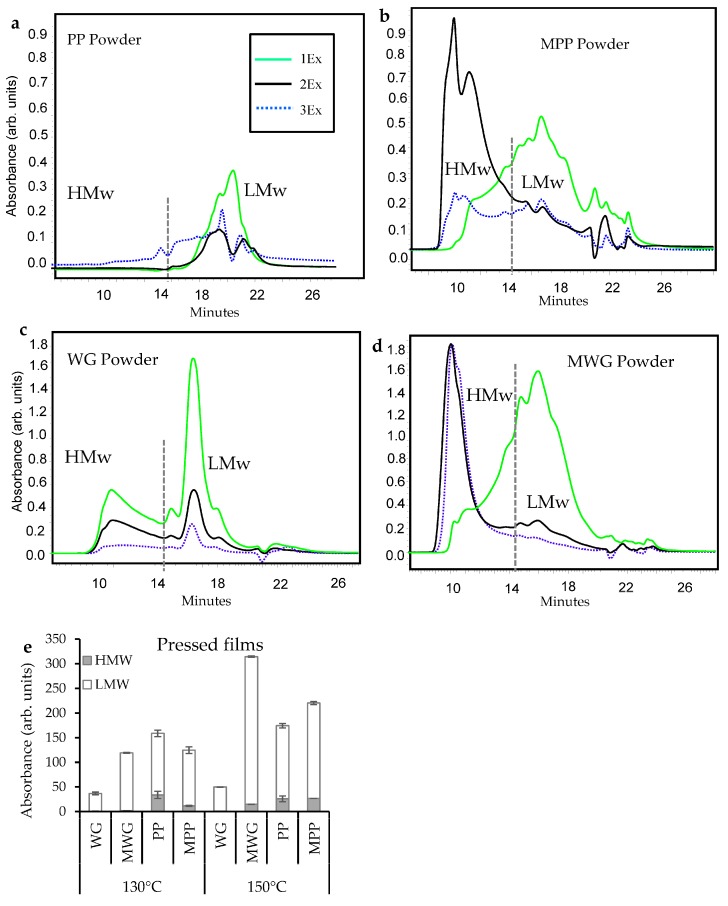
Representative chromatograms of protein molecular size distribution of potato protein powder (PP) (**a**), modified potato protein powder (MPP) (**b**), wheat gluten (WG) (**c**) and modified wheat gluten (MWG) powder (**d**), and the total protein extractability of films from the 3 extractions of non-modified and pH-modified proteins pressed at 130 °C and 150 °C (**e**).

In this study, SE-HPLC was used to assess protein polymerization behavior in the pH modified protein powders and high temperature processed films (Figure 1). The modified potato protein (MPP) and modified wheat gluten (MWG) powders showed greater amounts of extractable polymeric proteins (HMw and LMw) compared to the non-modified samples, PP and WG (Figure 1a–d). This was particularly evident for the HMw proteins extracted during 2Ex and 3Ex extractions (Figure 1b,d). The results indicated, that the alkaline pH induced unfolding in the studied protein and made the proteins more extractable. 

For protein films and composites processed at two temperatures, 130 and 150 °C, variation in protein extractability was observed. The processing of the protein powders into films at high temperature induced a higher degree of protein cross-linking and promoted the formation of larger protein aggregates and polymers, thereby decreasing their extractability during SE-HPLC analysis as has previously been reported in processed protein based materials [2,4,12,16,18,19,20]. In addition, possible interactions of the proteins studied with residual starches (wheat starch from wheat gluten fraction and potato starch from potato protein fraction) could have potentially occurred through non-covalent reactions such as, hydrogen and van der Waals-bonding between the protein and reactive hydroxyl groups from starch. Although those interactions were minor and did not substantially impact protein cross-linking.

For the pressed materials in this study an increase in protein extractability was seen for protein materials pressed at 150 °C, as compared to those pressed at 130 °C (Figure 1e). This increase in extractability in films pressed at the higher temperature (150 °C), was mainly due to an increase in LMw proteins being extracted as a consequence of protein degradation (except, for MWG, in which increase in HMw proteins at higher temperature was also observed) and lack of protein–protein interactions. In addition, proteins from films produced from the chemically modified proteins were in general more easily extractable, with the exception of MPP pressed at 130 °C (compare MPP with PP pressed at 130 °C; Figure 1e).

The protein extractability results clearly suggests that the basic pH-modification of protein powders contributed to a protein network unfolding, decrease in protein–protein interactions and de-polymerization of protein aggregates being present in the non-modified protein powder. The unfolding, decrease of protein–protein interactions and de-polymerization of the proteins after modification resulted in an increased extractability of the proteins. The fact that the extractability increased primarily as HMw proteins were extracted during 2Ex and 3Ex extraction steps (where sonication energy is used), suggests that primarily protein aggregates/polymers were affected by the pH adjustment (chemical modification). However, the majority of the protein extraction taking place at 2Ex and 3Ex indicated the presence of some of the cross-links, although with lower degree of intermolecular disulphide bonds, while hydrogen bonds and non-covalent protein interactions might still be in place [21,22]. 

Compression molding of the powder into films resulted in excessive cross-linking and re-formation of intermolecular disulphide bonds for all samples, as this has been previously reported for wheat gluten and potato protein materials [2,11,16,19,23]. In particular, most of the HMw proteins in all the hot pressed samples ended up in forming large polymers, which were not extractable in the films after pressing. It is also known from previous studies that compression molding of films under alkaline conditions leads to increased protein–protein interactions with the formation of disulphide cross-links in WG, hydrogen bonding in WG and PP and also the formation of isopeptide bonds such as dehydroalanin, lanthionine and lysinoalanine [7,16,24,25,26]. In addition, alkaline pH and the use of cross-linking enzymes, such as transglutaminase, for protein pre-treatment can further introduce new protein cross-linking opportunities, as has been shown in grass pea flour films [27]. Also, newly sourced transglutaminase species that have been shown to positively impact certain functional behavior of proteins in wheat dough [28,29] should further be explored in protein-based materials. 

In this study, the proteins were found to be less polymerized after hot pressing at 150 °C than at 130 °C, most likely due to protein degradation, with HMw proteins breaking into LMw fragments [8,23]. In terms of nano-structure for unmodified WG and PP samples pressed at 130 and 150 °C, few differences due to high temperature were observed in the main scattering broad peak, *d*_1_ and for the scattering distance *d*_2_, studied by SAXS, for both types of proteins (supporting information, Figure S1). For the PP film, the broad peak (*d*_1_) became “sharper”, while *d*_2_ intensity decreased with increasing temperature. While for the WG film, at 150 °C the scattering distance, *d*_2_, disappeared, indicated a less complex structural morphology compared to the previously observed structural morphologies in WG films pressed at 130 °C [5]. A possible explanation is a decrease in polymerization and an increase in protein breakdown as shown by SE-HPLC (Figure 1e). Regarding the PP film morphology, intensity of the *d*_2_ peak was higher at 130 °C than at 150 °C, indicating more structural complexity, although without any specific peak ratio [2]. This change in peak position and intensity may be affected by protein polymerization behavior, which was observed at different pressing temperatures. Regarding the MWG and MPP films morphology, the MPP showed a clear peak, *d*_1_, indicating similar structural morphology observed previously for the PP films pressed at 130 °C [2] (supporting information, Figure S2). While MWG showed *d*_1_ and *d*_2_, scattering distances, that can be referred to some undefined, though poorly hierarchically arranged structural entities (as compared to previously observed hexagonal arrangement of WG proteins). Thus, modification may not have considerably changed the nano-structure of MPP, while MWG nanostructure was not improved.

Previous study of WG has indicated that a less polymerized structure in the powder used for processing contributes to a higher degree of re-polymerization, chemical flexibility for proteins to interact and cross-link during processing, and the availability of chemically reactive sites for disulphide bond formation [5]. Although, the described effect could not be seen for the WG proteins in this study, indicating the alkali modification either leads to irreversible changes in the protein conformation (negative impact) or to chemical reactions not involving disulphide bond formation.

### 2.2. Protein Extractability in Pressed Composites

**Figure 2:**
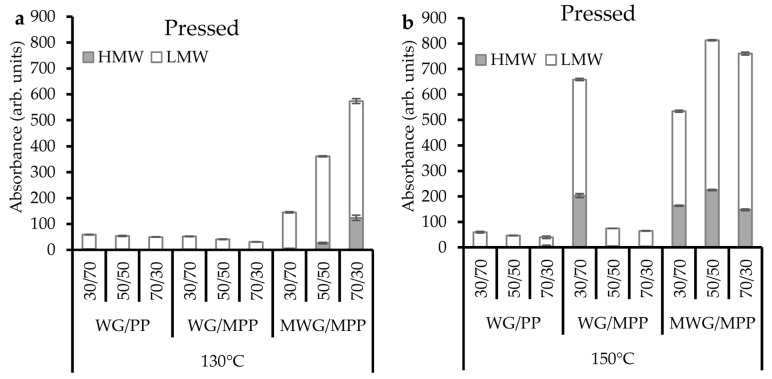
Total protein extractability of WG/PP, WG/MPP and MWG/MPP samples pressed at 130 °C (**a**) and 150 °C (**b**), as studied by SE-HPLC.

Among the pressed composites the highest protein extractability was clearly seen for the MWG/MPP samples at 150 °C, almost independent of the ratio of the two modified proteins within the composite (Figure 2). These results corresponded well with the high protein extractability found for the modified proteins pressed individually at 150 °C (compare Figure 1e), although protein extractability was even higher in the composites than in the pressed films of individual proteins. Thus, mixing of the two modified proteins did not contribute to increased protein polymerization as compared to protein polymerization for the modified proteins separately. Similarly, a high proportion of MWG (50 and 70%) in the composites pressed at 130 °C resulted in increased protein extractability as compared to the films from each of the individually modified proteins (Figure 2a compared with Figure 1). The extractability was also increased with the increase in temperature from 130 to 150 °C and with higher MWG content in the blend (Figure 2). An increased protein extractability with increase in MWG content (at 130 °C) could be due to the fact that MWG has a more complex protein molecular structure and chemistry, compared to PP, and was more denatured and depolymerized in the pH-modification process compared to PP (see MWG and MPP powder profiles; Figure 1b,d). Therefore, an MWG increase in the MWG/MPP blends (i.e., the 70/30 sample) resulted in a decrease in polymerization and lack of disulphide cross-link formation compared to 30/70 and 50/50 samples. However, using a 150 °C pressing temperature reduced the protein polymerization (increased protein extractability) for all MWG/MPP compositions compared to the individual modified proteins (compare Figure 1 and Figure 2b). This increase in protein extractability at higher temperature may be due to the lack of disulphide and irreversible cross-links between the protein chains and also due to breakdown of HMw protein into LMw fragments.

For the composites based on non-modified proteins (WG/PP), such an increase in protein extractability (as seen for modified samples) was not seen (compare Figure 1 and Figure 2); instead a slight decrease in protein extractability was observed, especially at high WG ratios (70/30). The non-modified (as received) WG and PP have a low extractability due to the formation of HMw protein aggregates during their industrial processing [2,5]. Therefore, their further processing at high temperatures favored the formation of non-reducible protein cross-links and also incorporation of the LMw fragments in larger protein networks, thus reducing their extractability [8,22,30]. This suggests that composites of non-modified proteins produced in this study did show reduced protein extractability as reported for individual protein based materials produced previously [2,19]. 

As for the WG (non-modified) composites with different ratios of MPP, the protein extractability was in a similar range to that of WG/PP films at both pressing temperatures (compare Figure 2 and Figure 1) except for WG/MPP 30/70 at 150 °C with surprisingly higher amounts of extractable proteins. Hence, higher pressing temperature (as 150 °C) increased protein polymerization and decreased extractability when ≤ 50 *wt*.% of MPP was used. No such effect of temperature and amount used of MPP was seen in the samples pressed at 130 °C, suggesting that high temperature may be one of the main driving factors in determining the protein polymerization. 

Interestingly, the lowest protein extractability (highest degree of protein polymerization) was found for the 70/30 WG/MPP composite film at 130 °C, and for the 70/30 WG/PP film at 150 °C (Figure 2). Thus, to increase protein polymerization in WG based films, modified potato protein (MPP) is beneficial at 130 °C, while non-modified PP is beneficial to have at higher temperature. Increased protein polymerization in 70/30 WG/MPP could be explained by a higher degree of protein cross-linking of WG at 130 °C as has been reported in previous studies [5,19,31]. However, an increased protein polymerization in WG/PP 70/30 sample at 150 °C suggests formation of new protein–protein interactions in a composite, because the extractability of these blends decreased slightly more when compared to both non-modified proteins pressed at 150 °C.

### 2.3. Effect of Chemical Modification on Secondary Structure of Proteins

**Figure 3:**
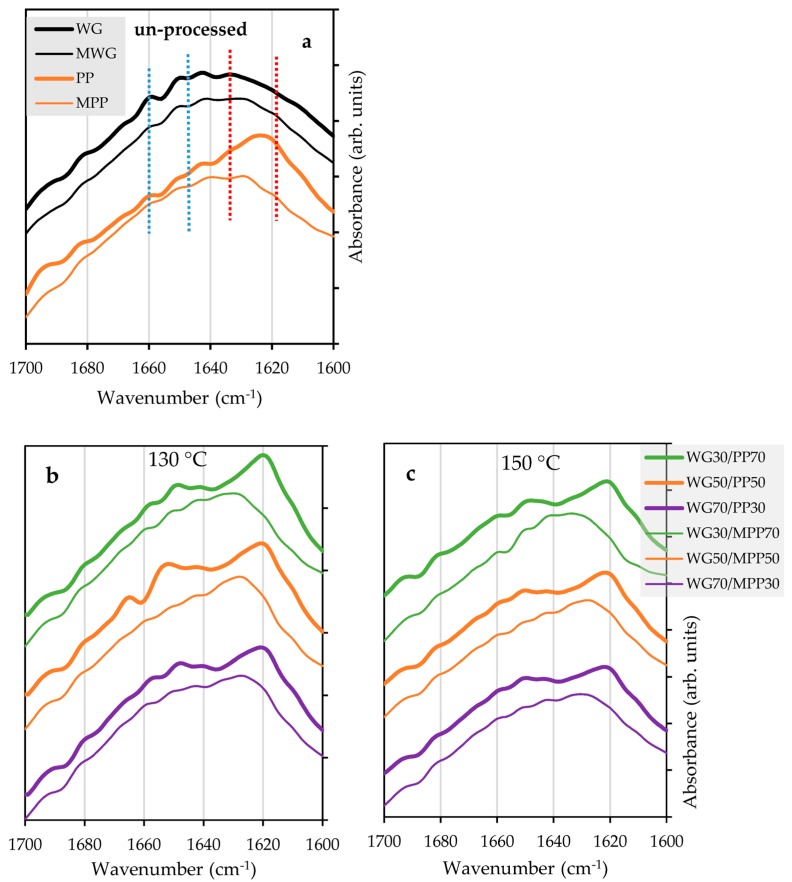
FT-IR spectra of individual protein powders and pressed films at 130 and 150 °C. WG, MWG, PP and MPP powders (**a**), pressed samples of WG/MPP blends along with controls (**b**,**c**).

As shown in previous studies [5,10,12,31,32,33], the non-modified WG powder showed relatively low amounts of β-sheets, as indicated by a flat shoulder in the amide I region (1620–1625 cm^−1^), while peaks at 1660, 1650 and 1641 cm^−1^ verified the presence of α-helices, α-helices and random coils, and unordered structures, respectively (Figure 3a). Chemical modification of the WG protein (MWG sample) resulted in a slight decrease in the intensity of peaks in the region 1640–1660 cm^−1^, indicating that some of either α-helices, α-helices and random coils, or unordered structures became involved in formation of strong hydrogen bonded β-sheets (Figure 3a). Thus, FT-IR data indicate formation of β-sheets at the expense of either α-helices, α-helices and random coils or unordered structures, may indicate development of novel interactions (presumably via hydrogen bonds, protein-protein and peptide–protein interactions [34]), although not verified by SE-HPLC results (Figure 1).

The FT-IR spectra of PP showed a very distinct and high intensity peak at 1622 cm^−1^ verifying the presence of strongly hydrogen-bonded β-sheets. The formation of these protein structures are known to originate from the harsh treatment of the PP during industrial extraction with the use of high temperature and acidic processing conditions promoting a high degree of cross-linking [2]. The high degree of cross-linking in PP was also supported by the SE-HPLC data, showing low extractability of PP powder (Figure 1a). Furthermore, results of the SE-HPLC were supported by FT-IR data for the MPP powder sample, in general showing a lower amount of strongly hydrogen-bonded β-sheets (less aggregated structure) as reported in previous studies [4,34,35].

The pressed WG/PP blended films resulted in FT-IR spectra with a clear and high intensity peak at 1620 cm^−1^ and also several well defined peaks in the 1640–1660 and 1622 cm^−1^ region (Figure 3b,c), thereby showing structural features resembling both single powders used in the blend (Figure 3a) and indicating presence of strongly bonded β-sheet interactions, α-helices, α-helices and random coils, and unordered structures [2,5]. Including MPP in the blends (WG/MPP) for compression molding resulted in FT-IR spectra with reduced intensity and a shift to lower frequency of the major β-sheet peak (especially at 150 °C when the shoulder also became broader), and a decrease in intensity of the peaks in the region of 1640–1660 cm^−1^ (Figure 3b,c). In general protein aggregation has been correlated to an increased amount of strongly hydrogen-bonded β-sheets contributing to ordered conformation and improved tensile strength [2,4,7,10,12,31,32,33,34,36,37,38]. However, the lowest extractability of the proteins (indicating protein polymerization) as resolved by SE-HPLC results (Figure 2) was found for WG/MPP 70/30 at 130 °C and WG/PP 70/30 at 150 °C, not corresponding with blends showing the most intense peak for β-sheets by FT-IR (Figure 3b,c). These results suggest that the protein polymerization behavior and relationships with structural properties seems to be more complicated for compression molded composites than for individual proteins, especially while modifying of the chemical structures of the proteins used in the blends.

FT-IR spectra of MWG/MPP composites showed more differentiation between the ratios of proteins, where the strongly hydrogen-bonded β-sheets related structural peak was more pronounced at higher PP ratio and lower temperature (supporting information, Figure S3), corresponding to lower protein extractability by SE-HPLC (Figure 2). This might indicate that differences in protein polymerization were too limited among WG/PP and WG/MPP blended compression molded films to be differentiated by FT-IR in most cases, and basically only differences between separate blended protein components were seen. Thus, only for differences in protein polymerization behavior of MWG/MPP blends were large enough to be differentiated by FT-IR as also indicated by SE-HPLC, and corresponded to the characteristic relationship of SE-HPLC, FT-IR and functional properties data [12,31].

### 2.4. Effect of Protein Modification on Mechanical Performance of the Pressed Composites

**Figure 4:**
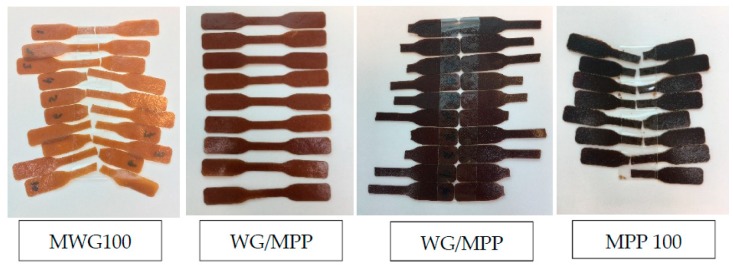
Representation of the range of colors of films and composites MWG100, WG/MPP 70/30, WG/MPP 30/70 and MPP100 pressed at 130 °C.

Compression molding of WG, MWG, PP, MPP and their blends, yielded samples of different colors as can be seen in Figure 4. High WG and MWG content generally resulted in a sample with light brown color (Figure 4, MWG100 and WG/MPP 70/30), while high PP and MPP content generally resulted in samples with dark brown (WG/PP 30/70) or almost black in color (MPP100), which can influence the composites’ attractiveness and end-use. It is however an easy task to incorporate a pigment or dye to obtain a desired color (excluding white color). WG and PP samples showed opposite tensile performance, WG having lower strength and E-modulus but higher extensibility (elongation at break) than PP. Thus, comparing these two protein types, the tensile strength correlated positively with protein extractability. This observation was different to many previous studies, where a higher degree of cross-linking yielded higher strengths [2,5,19,22,39,40]. Here, the WG proteins most likely have a higher ability to cross-link (due to their more complex molecular structure characteristics and specific polymerization properties [8,22]) during pressing than the PP as SE-HPLC results indicate, although FT-IR shows a higher degree of β-sheets in the pressed PP which might explain their higher maximum stress and E-modulus [2].

**Figure 5:**
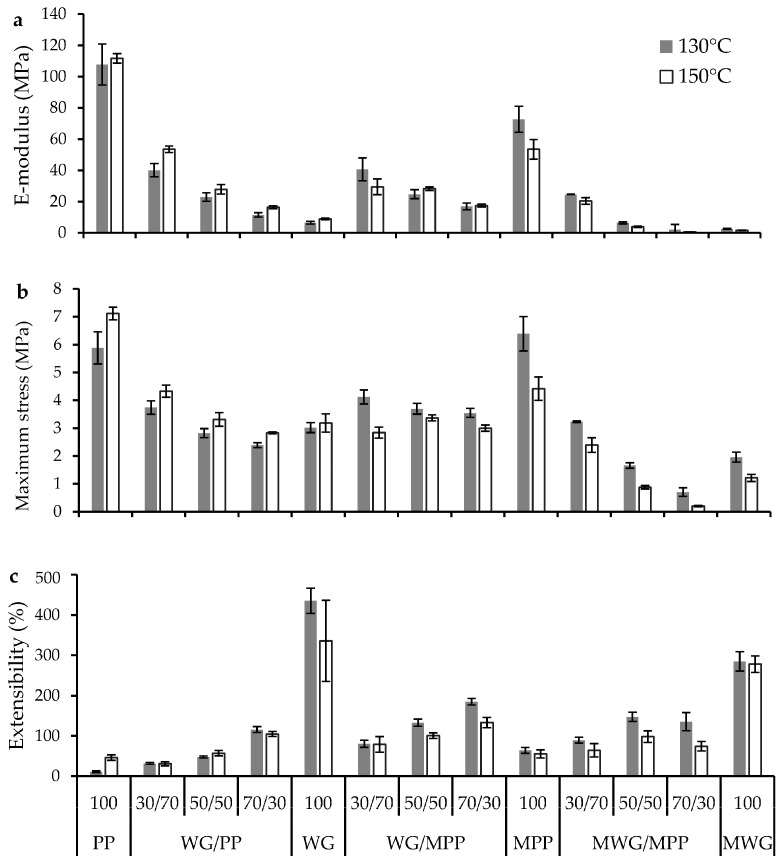
Mechanical properties of individual protein films and WG/PP, WG/MPP and MWG/MPP blends pressed at 130 and 150 °C; (**a**) E-modulus, (**b**) maximum stress and (**c**) extensibility.

Modification of WG (MWG) resulted in lower maximum stress and E-modulus and reduced extensibility compared to WG for both evaluated pressing temperatures (Figure 5). Modification of the PP (MPP), resulted in a decrease in E-modulus for samples pressed at both temperatures and maximum stress decreased at 150 °C while the extensibility increased particularly at 130 °C (Figure 5). SE-HPLC data indicated an increase in cross-linking at 130 °C, although a decrease in β-sheet content was shown by FT-IR, which might explain the improvement in the extensibility and decrease in E-modulus for the MPP as compared to PP. In addition, a previous study reported an increase in extensibility and a decrease in modulus for PP modified with NaOH, when pressed with 30% glycerol [4]. Thus, modification of PP prior to processing (e.g., hot pressing) leads to improved tensile properties but also contributes to opportunities for using lower processing temperatures than was used in previous studies (≥ 150 °C) [2], thereby saving energy input during the production process.

The pressed composites of WG and PP resulted in tensile properties generally as expected from the ratios of the individually pressed proteins (Figure 5). In addition, pressed WG/MPP samples showed tensile properties generally between those of the individually pressed protein samples, although minor differences were seen among the samples. Furthermore, as also indicated by comparing FT-IR and SE-HPLC results, differences in the protein cross-linking obtained by SE-HPLC could not explain the differences in tensile properties between the WG/PP and WG/MPP blends.

The most rubber-like properties were observed for the pressed MWG material (lower stiffness than WG), which also resulted in that the MWG/MPP blends were with the most rubber-like properties (low modulus but still with appreciable extensibility) (Figure 5), thereby correlating with SE-HPLC and FT-IR data. It is noteworthy that the higher pressing temperature (150 °C) leads to lower stiffness, strength and extensibility for the modified materials and their blends (MWG, MPP and MWG/MPP) (Figure 5).

## 3. Materials and Methods

### 3.1. Wheat Gluten and Potato Proteins

Wheat gluten with protein content of 77.7 *wt*.%, starch 5.8 *wt*.%, moisture 6.9 *wt*.% and fat 1.2 *wt*.%, was purchased from Lantmännan Reppe AB, Lidköping, Sweden. Commercial PP was supplied by Lyckeby Starch AB, Kristianstad, Sweden. The potato protein content was 82.2 *wt*.% (Dumas method, Flash 2000 NC Analyser, Thermo Scientific, USA, NX 6.25) and moisture content of 8.1% (dry basis, dried at 105 °C for 3 h). Glycerol (purity 99.5 *wt*.%, 0.5 *wt*.% water) was supplied by Karlshamns Tefac AB, Karlshamn, Sweden.

### 3.2. Protein Modification

Potato protein powder, 50 g was slowly dispersed in 600 mL distilled water under stirring. The suspension was adjusted to pH 10 by addition of a 5 M NaOH solution, followed by stirring for 30 min at 75 °C (± 3 °C). The PP was well dispersed in these conditions forming a dark brown suspension. The PP suspension was then lyophilized and ground to powder using an IKA A10 grinder (IKA, Germany) and was designated as modified PP (MPP) in this study. WG was modified in the same way as the PP above and was designated as modified WG (MWG). Protein blends were prepared by dispersing various ratios of WG and PP in water followed by the pH modification step as described for the separate protein sources. Thereafter, MWG/MPP blend were lyophilized and ground to obtain powder for further sample processing.

### 3.3. Sample Preparation and Compression Molding

Powders from individually modified PP and WG proteins were mixed with glycerol, (70 *wt*.% protein and 30 *wt*.% glycerol) prior to compression molding and later pressed into films. Each type of protein was pressed into two films, one at 130 °C and another one at 150 °C. For blends, different ratios of WG/PP and WG/MPP (30/70, 50/50 and 70/30) were manually mixed with glycerol, (70 *wt*.% protein and 30 *wt*.% glycerol) prior to compression molding. The sample compositions are presented in Table 1. The prepared individual protein films and blends were then placed in a 0.5 mm thick aluminum frame with a 100 × 100 mm opening to control the size and thickness of the film. The film and blends were then molded for 5 min at 200 bar in a hydraulic press (Polystat 400s, Servitech, Germany) between pre-heated aluminum plates with poly(ethylene terephthalate) release films. From each of the blends two films were compression molded, one at 130 °C and another one at 150 °C. Pressed films were removed from the hot press and left to cool between two room temperature aluminum plates.

### 3.4. SE-HPLC to Assess Protein Polymerization in Processed Composites 

The soluble amount of protein and protein size distribution of the protein powders, compression molded samples and composites were examined in this study. For this analysis, films were cut into approximately 0.2 mm pieces using a scalpel. Thereafter, 16.5 mg (± 0.05 mg) of each sample was added to 1.4 mL buffer solution (0.5% SDS, 0.05 M NaH_2_PO_4_, pH 6.9) in a Eppendorf tube (1.5 mL). To obtain the first extraction (1Ex) tubes were vortexed for 10 s (Whirli Vib 2, Labassco, Sweden) and then shaken for 5 min (IKA-VIBRAX VXR, IKA, Germany) at 2000 rpm. The tubes were then centrifuged (Legend Micro 17, Sorvall, Germany) for 30 min at 12,500 rpm and the supernatant was collected in HPLC vials. For the 2nd extraction (2Ex), extraction buffer (1.4 mL) was added to the pellet from 1Ex and then sonicated for 30 s at an amplitude of 5 microns using a Sanyo Soniprep 150 Ultrasonic Disintegrator (Tamro, UK) and thereafter centrifuged for 30 min and the supernatant collected in HPLC vials for analysis. The third extraction (3Ex) was similar to 2Ex, the pellet of 2Ex was used with sonication intervals of 30 + 60 + 60 s, to avoid overheating, samples were left to cool at room temperature between each sonication interval. All three extractions (1Ex, 2Ex and 3Ex) were analyzed with a Waters 2690 Separation Module connected to a Waters 996 Photodiode Array Detector (Waters, Millford, MA, USA).

An SE-HPLC column (Biosep-SEC-S 4000, Phenomenex, Torrance, CA, USA) was used for protein size distribution determination. For each sample, 20 µL was injected onto the column at an isocratic flow of 0.2 mL/min (50% acetonitrile, 0.1% TFA; 50% H_2_O, 0.1% TFA). Chromatograms were obtained at 210 nm and integrated using Empower Pro software (Waters, USA). The chromatograms were divided into two sections depending on the elution time of the proteins and total area was calculated. The high molecular weight (HMw) proteins were eluted between 7–14.5 min and low molecular weight (LMw) between 14.5–28 min. HMw are referred to as polymeric proteins, and LMw as smaller molecular size proteins.

### 3.5. Tensile Testing 

For mechanical testing the films were conditioned for at least 48 h at 23 °C and 50% relative humidity and then cut into dumbbell shaped samples (ISO 37 type 3, Elastocon, Sweden) and thereafter tested in the same conditions. Prior to testing the thickness of all the samples was measured at 5 different points (Mitutoyo IDC 112B) in the test area and the average was used for calculations. A clamp separation distance of 40 mm, crosshead speed of 10 mm/min and a 500 N load cell was used to test all samples on an Instron 5566 universal testing machine with a Bluehill software (Instron AB, Danderyd, Sweden).

### 3.6. Fourier Transform Infrared Spectroscopy

All the samples were dried for at least 72 h in a desiccator over silica gel prior to the analysis. FT-IR spectroscopy was carried out on all films and powders using a Spectrum 2000 FT-IR spectrometer (Perkin-Elmer inc., Norwalk, CN, USA) equipped with single reflection ATR (Golden Gate, Speac Ltd., USA). Data was Fourier self-de-convoluted using the Spectrum software with γ=2 and a smoothing factor of 70%.

## 4. Conclusions

Chemical modification of plant proteins, through heating at a basic pH, results in a protein with a decreased amount of intermolecular disulphide bonds and thereby a less cross-linked structure, as shown here for both WG and PP. Cross-linking of the proteins in the starting powder, when producing bio-based materials, largely impacts the properties of compression molded materials both in terms of opportunities for the proteins to further cross-link and form new bonds and structures during processing. From our previous study, mild protein extraction of WG has been shown to contribute with less cross-linked protein powder which when hot compression molded into films resulted in improved tensile properties (E-modulus and maximum stress) [5]. However, a less cross-linked structure in the protein starting material does not necessarily contribute to improved properties of processed bio-based materials. Here, a decrease in cross-linking of proteins by chemical modification, improved cross-linking (during temperature pressing) and tensile properties (E-modulus and maximum stress) of the material of PP but not for WG. Thus, using basic pH for modification of WG must contribute to irreversible changes in the protein conformation/structure and/or to chemical reactions not involving disulphide bond formation, which reduces the opportunities for the proteins to re-cross-link when processed. For PP, instead the chemical modification through basic pH contributed to the breaking of disulphide bonds which were created during industrial production of the PP starting material (the powder).

A major advantage with the chemical modification of PP was that it enabled the production of films with improved properties at a pressing temperature as low as 130 °C, thereby contributing to a lower environmental foot-print due to a reduction of energy use.

**Figure 6:**
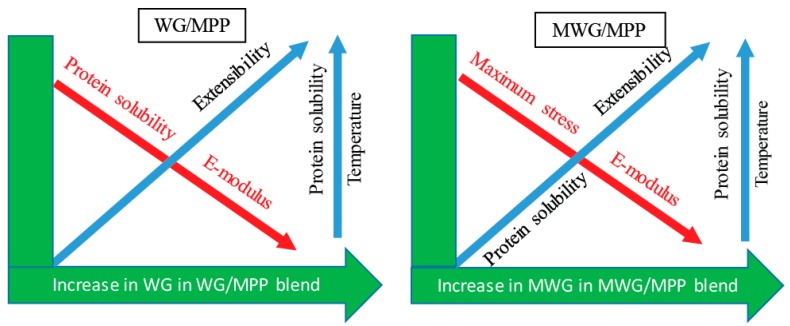
Schematic diagram of summarized effects of temperature and composition of the blend on protein polymerization and mechanical properties.

Thus, chemical modification of proteins for bio-based material production on an industrial scale may potentially help to widen the functional properties and improve the socio-economic value of the product (especially in the case of PP). Figure 6 provides a schematic summary of the important findings of this study.

## Figures and Tables

**Table 1:** Compositions of the blends of WG, PP, MWG and MPP.

Type	Abbreviation	WG (*wt*.%)	PP (*wt*.%)	MWG (*wt*.%)	MPP (*wt*.%)
Controls	WG/PP 30/70 WG/PP 50/50 WG/PP 70/30	30 50 70	70 50 30	--- --- ---	--- --- ---
Only PP Modified	WG/MPP 30/70 WG/MPP 50/50 WG/MPP 70/30	30 50 70	--- --- ---	--- --- ---	70 50 30
MWG/MPP Modified in Composite	MWG/MPP 30/70 MWG/MPP 50/50 MWG/MPP 70/30	--- --- ---	--- --- ---	30 50 70	70 50 30

## Supplementary Materials

Supplementary materials can be found at http://www.mdpi.com/1422-0067/20/1/58/s1.

## Abbreviations

Ex	Extraction
FT-IR	Fourier Transform Infrared Spectroscopy
HMw	High molecular weight proteins
LMw	Low molecular weight proteins
MPP	Modified potato protein
MWG	Modified wheat gluten
PP	Potato protein
SE-HPLC	Size Exclusion High Performance Liquid Chromatography
*T*_g_	Glass transition temperature
WG	Wheat gluten
