# Supplementary material for: Impact of pH Modification on Protein Polymerization and Structure–Function Relationships in Potato Protein and Wheat Gluten Composites
Source: Int J Mol Sci. 2018 Dec 24;20(1):58. doi: 10.3390/ijms20010058 (PMC6337652; doi:10.3390/ijms20010058)
Supplement: Supplementary file 1 [file ijms-20-00058-s001.pdf]

Supplementary information

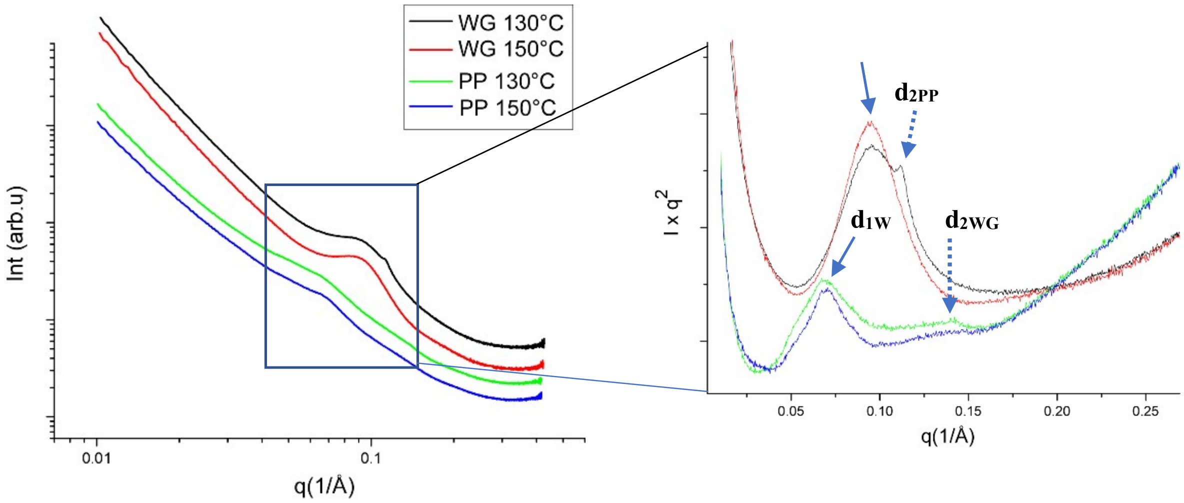

Figure S1. SAXS curves of WG and PP pressed one at 130 and another one at 150 °C. Curves are shifted vertically for clarity and I x q^2 plot is drawn to enhance the behavior of d_1 and d_2 peaks.

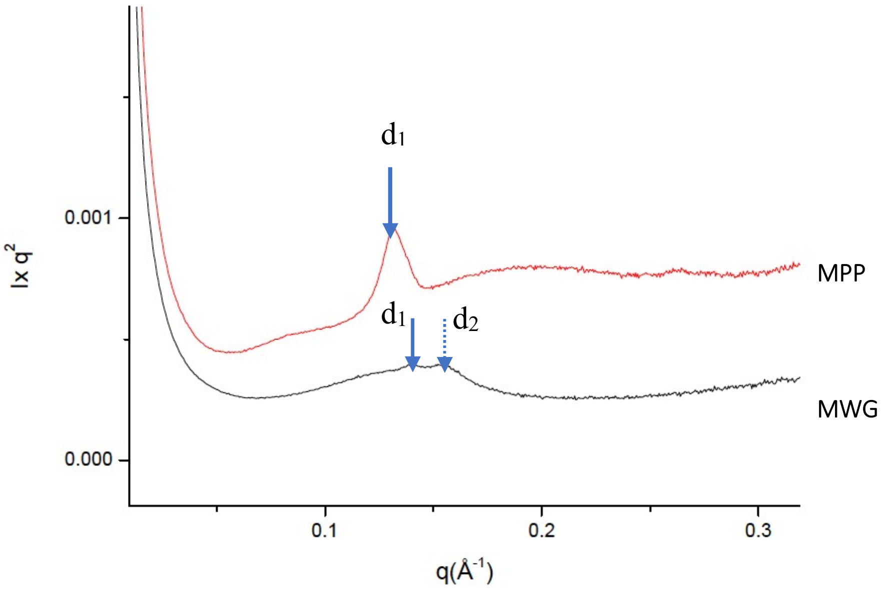

Figure S2. SAXS curves of MWG and MPP films pressed at 130°C. I x q^2 plot is drawn to enhance the behaviour of d_1 (dotted and intense arrows) and d_2 (intense arrow) peaks.

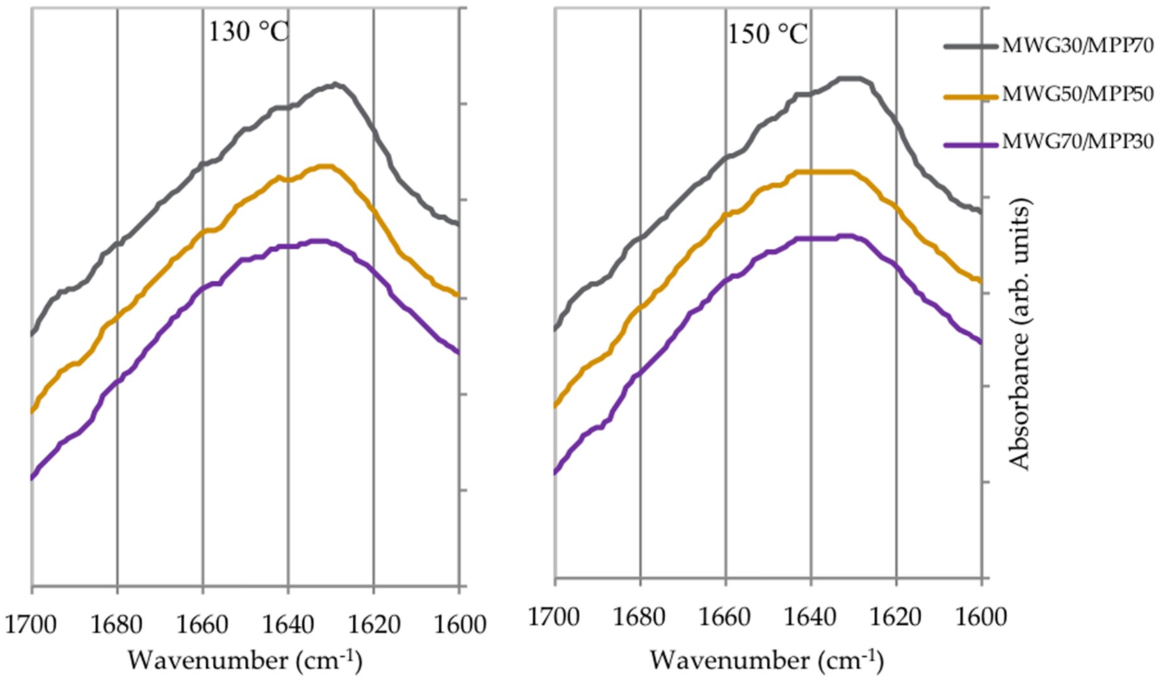

Figure S3. FT-IR spectra of MWG/MPP blends, one pressed at 130 and another one at 150 °C.
